# The Community Health Experience Model—value generation from person-centered health transaction network

**DOI:** 10.1186/s40985-018-0105-8

**Published:** 2018-10-01

**Authors:** Zoltán Lantos, Judit Simon

**Affiliations:** 1 Corvinus University of Budapest and Jill Health Guide ApS, Copenhagen, Denmark; 2 Corvinus University of Budapest, Fövam ter 8, Budapest, 1093 Hungary

**Keywords:** Value-based healthcare, Person-centeredness, Co-creation, Health experience, Medication management, Értékalapú egészségügy, egyénközpontúság, kokreáció, egészségélmény, terápiamenedzsment

## Abstract

**Background:**

Network society is creating new opportunities for value generation in all areas of our lives: new collaborative methods and tools are increasingly available for use by closely connected individuals and organizations. The stakeholders of the health ecosystem are potential winners of this networking process as a consequence of the increase in knowledge about health value generation supported by teamwork and collaborative approaches in this field.

**Case Presentation:**

In this paper, we focus on the transactional nature of health value generation networks. First, we analyze the transactions in the networks. We then propose a design structure—the Community Health Experience Model—for effective person-centered health value generation networks. In the second phase of the work, we describe how the system design of the complete transaction network was tested in a real-life pilot environment focusing on fall prevention in individuals with osteoporosis.

As a result of the network-based collaborative service approach, fall risk decreased by 11.8% and the number of falls decreased by 4.5% within 3 months. Regarding the major health experience outcomes, self-evaluated condition-specific health literacy improved from 7.85 to 8.26 (an improvement of 0.41), while self-evaluated condition-specific self-management capability changed from 7.25 to 8.06 (0.81 improvement).

**Conclusions:**

In conclusion, the proposed Community Health Experience Model is a novel and promising approach to designing the structure of more effective and efficient health services and collaborative networks.

**Electronic supplementary material:**

The online version of this article (10.1186/s40985-018-0105-8) contains supplementary material, which is available to authorized users.

## Összefoglaló

**A kutatás háttere:**

A hálózatos társadalom életünk minden területén új lehetőségeket kínál az értékteremtésre az internet által is szorosan összekötött egyének és szervezetek számára. Az egészség-ökoszisztéma szereplői és érintettjei valószínű nyertesei ennek a hálózatosodásnak az egyre gyarapodó tudásunknak köszönhetően, amely az egészségérték csapatmunkában és együttműködéssel történő megteremtéséhez kötődik.

**Az esettanulmány bemutatása:**

Munkánkban az egészségértéket megteremtő hálózat tranzakcióinak sajátosságaira összpontosítottunk. Először elemeztük az egészséget megteremtő hálózatokra jellemző tranzakciókat, majd leírtuk a hatékony egyénközpontú egészségérték-teremtő hálózatokra javasolt működési struktúrát, a Közösségi Egészségélmény Modellt. Az elvégzett munka második szakaszában a teljes tranzakciós hálózati rendszer-designját teszteltük valós életbeli körülmények között, csontritkulásos egyének esésmegelőzésére összpontosítva.

A hálózatos együttműködéseken alapuló szolgáltatásstruktúra eredményeként az eséskockázat 11,8%-kal csökkent, míg az esések száma 4,5%-kal csökkent három hónap alatt. A két kiemelten vizsgált egészségélmény mutató, az önértékeléssel mért egészségműveltség tízfokozatú skálán 7,85-ről 8,26-ra nőtt (0,41 növekedés), míg az önértékeléssel mért állapotspecifikus önmenedzsment képesség 7,25-ről 8,06-ra (0,81 növekedés).

**Következtetések:**

Az eredmények alapján a Közösségi Egészségélmény Modell ígéretes új megközelítést nyújt a hatékonyabb és hatásosabb egészségszolgáltatások és együttműködő hálózatok megtervezéséhez.

## Background

### Health and experience

#### The experience economy

Personal experience is currently the focal point of the economic system and is achieved through relationships at a community level. The present era was preceded by ones of production and services [1] and, as a result, society’s views about value exchange have also changed fundamentally. Philip Kotler, together with Achrol [2], calls attention to the fact that in the new social order, we are required to think entirely differently about marketing, that is, about value exchange. At the individual level, experience is the purpose of exchange; at the community level, relationships are becoming dominant and are the main driving force of value interactions; in the globalizing society, however, the main task is to define individual and social responsibilities within the framework of this new society.

In this paper, we summarize the result of our extensive customer-centric economic work focusing on health, mostly using the theoretical framework of marketing and behavioral science. We describe an economic framework of potential use in the description and management of the health-specific customer journey and the corresponding outcomes that result in improvements in customer value. A condition-specific real-life pilot is also described with initial findings.

#### The new approach to healthcare

Healthcare and health services are undergoing a paradigm shift from being medical science- and medical doctor-oriented to becoming person-centered [3].

**Fig. 1:**
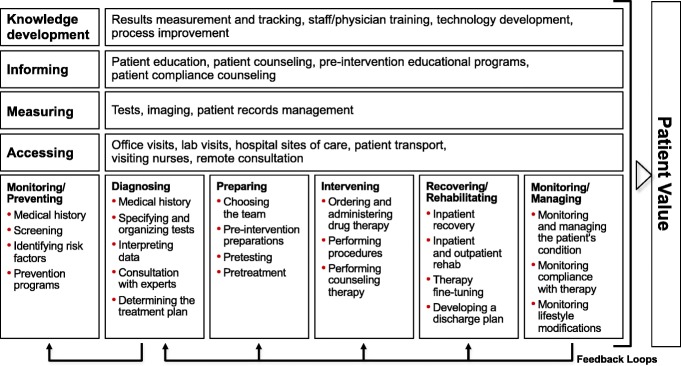
The Care Delivery Value Chain [5]

Although medical science has recognized the concept of patient-centered care methodology for more than 20 years [4], the institutional organizational framework for everyday practice has not been able to support the theory. The economic model of the Care Delivery Value Chain (Fig. 1) [5] ignited a cascade of substantial changes in healthcare.

New healthcare models expand the focus of care from medical intervention (i.e., problem solving) to the complete process of health value generation—using the medical term, “continuum of care”—based on health maintenance and health management. Thus, the contributions of healthcare providers are described in terms of longer, well-defined processes, suggesting the necessity of extensive teamwork. As a result, new players are becoming involved in healthcare teams such as patient navigators, health coaches, hospitalists, directors of patient experience, care transition coordinators, and physician champions [6]. According to a recent analysis, “the organizations that have been shifting their strategies toward value-based care generally share certain advantages: financial stability, positive relationships with physicians, advanced information systems, and (often) affiliation with a health plan” [7]. The value-based approach puts the patients’ interests—convenience, wholeness, their own contributions, understanding, and tranquility—at the center of the care service process, instead of relying on a medically designed, technology-driven, functional, correctional approach.

According to Ekman et al., person-centered care refers to a type of care whereby the care provider focuses on the needs and resources of the patient and can be defined as the co-creation of care by patients, their family, informal caregivers, and health professionals [8, 9], while personalized care planning includes the following components [10]:

1. Patients and clinicians identify and discuss problems caused by or related to the patient’s condition(s), giving due consideration to both clinical tests and treatments and the practical, social, and emotional effects of their condition(s) and treatment(s) on their daily lives.

2. They then engage in a shared decision-making process involving goal setting and action planning, focused on determining priorities, agreeing about realistic objectives, solving specific problems, and identifying relevant sources of support.

3. The agreed plan is documented and followed up.

The person-centered care approaches using these three components generate improvements in care quality and specific outcomes [9, 11, 12].

Although real patient-centeredness as a major characteristic of healthcare systems is still seldom found in the real world, there is growing evidence of the success of the value-based approach based on measureable outcomes [13]. Reengineered care processes in some hospitals and clinics have enabled care teams to substantially decrease intervention-specific mortality and condition-specific harm. The increase in knowledge and evidence about the characteristics and elements of a sustainable healthcare system bring the realization of the value-based approach much closer [14].

The value-based approach to healthcare services has important implications at the system level as well. The continuum of care for any condition is a highly complex process consisting of numerous activities often performed by different market players. Following the logic of the value chain, each single activity that contributes to the continuum of care should organically connect to both the preceding activity and the subsequent one, thereby playing an appropriate role in distributing available resources.

The complete set of condition-specific continuums of care, together with each of the activities that serve for their realization along with the corresponding actors and required resources, form a service ecosystem that is dynamic and evolving [15]. The healthcare ecosystem has four different layers of cooperation from the service ecosystem perspective, the micro level with individuals, the meso level with providers, the macro level with professional organizations, and the mega level with enablers and regulators [16], corresponding to the Health in All Policies [17] approach that is designed to support system-wide patient-centered improvements.

#### The health ecosystem

Besides the revision of the care processes, our understanding of health has also changed at the level of the individual. We now need to look at personal health as a result of the constant everyday activity and related transactions of the individual. Remaining healthy takes effort, and individuals require energy for this [18]. There are two major energy sources: social networks with their intense interpersonal relations [19], and spirituality [20]. For the maintenance of health, individuals need different competencies: (a) those they know and are able to use, also referred to as health literacy [21]; (b) those which are purchased in the form of products and services; and (c) those individuals obtain through different non-financial transactions, predominantly through online social networks. The complete conglomeration of the above competencies and transactions constitutes the health ecosystem [22]. The public healthcare ecosystem is one part of this health ecosystem, accounting for between 37 and 61% of value in different countries [23].

#### The health experience

In a human-centric society in which experiences and relationships are the core elements of value creation, health is also seen from a unique perspective. Primarily, health refers to health-related experiences, but in special cases, going through an experience of recovery can foster personal value [24]. Since personal experience has become the dominant feature of our society regarding individual values, health is increasingly seen as personally lived well-being—“a health experience”—rather than the selected functions of a human being compared with defined metrics.

Individuals need to make continuous efforts to permanently “live their health” through personal health experiences, and they meet with numerous health competences that would support these efforts, only some of which they take advantage of. “Competence” here constitutes the fundamental unit of exchange or transaction as the sum of the specialized skills and knowledge of a manufacturer or a service provider [25]. Every competence—product or service—that supports an individual’s efforts toward a positive personal health experience is worth exchanging; on the other hand, an individual will always avoid all competences that do not support or increase their efforts. Therefore, the foundation of the good relationship that is needed for lasting value creation is the support of personal “health efforts” involving personal care, attention, and concern [26].

It is also reasonable to say that health is an outcome of social activities. This is partly because of socially created knowledge that will always be more comprehensive than that of the individual and partly because of the contribution a community can make to healthy living.

In line with general marketing theories (that is, in line with the value-exchange logic), products or services give individuals an opportunity to create health in an exchange process. Such exchanges are based on knowledge and competences that are utilized in “health co-production,” which industry experts call health co-creation [27]. The traditional organizational structure of healthcare services and the healthcare system can also create health value, but only with the engagement of the customer: the patient.

Studies among patients [28, 29] reveal that a variety of solutions are adopted to create health during the health co-creation process, and competences are selected to contribute to recovery and/or health-related experience from a very wide range. In this process, healthcare is usually only one factor, and often not even the most important one. Consideration of the ability of healthcare to generate the health experience and its supporting environment shows why its importance is gradually declining and marginalization increasing within the health ecosystem [30–32].

Individuals use their own competences, which are defined according to their specific health behaviors, and undertake the corresponding transactions. The more competences the individual uses in a personally appropriate manner, the more health they are able to create for themselves. Healthy living as an economic phenomenon involves a life-long process of competence transactions, a sequence of activities that is coupled with life-long learning [33]. Individual decisions about the use of competences are the result of personal health experiences. These health experiences are developed during the use of the competence or are generated by the result of use. Thus, according to the human-centered approach to health services, personal health value is a combination of medical performance and personal health experience [34].

## Case Presentation

### Human-centered healthcare

Although the Care Delivery Value Chain provides clear direction for change in healthcare, system-level transformation encounters numerous obstacles since a strong medical focus and silo-based institutional structures [35, 36] hinder system-wide change management.

One of the most important organizational changes in management involves the role of medical doctors. From “lone healing heroes”, these agents need to become team members and team leaders [37]. One major obstacle to this transformation is a method of practicing that is based on the prompt and complex decision-making that is usually done by physicians. It also means that the majority of such decisions are made at a high level of competency, although a substantial proportion of them could be made at a lower level.

Understanding the relevant economic and individual needs, some organizations have taken health management into their own hands and developed a system of individually designed health provisions for some of the more prevalent medical conditions [38]. Other successful initiatives have concentrated on the team-based community approach of primary care [39]. The implementation of health co-creation or co-production approaches have given impetus to the development of person-centered health services [27]. The co-creation practices typology that demonstrates how these practices are different in terms of shaping the health ecosystem [16] gives further practical guidance for service design and patient-centered service integration efforts.

In parallel with the development of service design, methods for the systematic analysis of patients’ voices have also been improved [40]. Further analysis has been undertaken about the role of shared knowledge and experiences using a person-centered eHealth approach [41].

The focus on patient and human centeredness and the integrated implementation of healthcare services make it necessary to specify and design the role a health manager or a therapy manager should play. The task of the former is to ensure that for the “health consumer,” the most appropriate services and treatments are selected from those that are available. A health manager should help customers to live a healthy life and to come to decisions regarding healthcare services and financial options in an optimal way [42]. Person-centered health planning is able to emphasize patients’ own resources that strengthen their self-efficacy and make care more effective [43]. The patient-centered model of various forms of care, services, and public health programs suggests that, without help and support, patients and health consumers face an extremely difficult situation when making health-related decisions. Very few health consumers are able to select all the necessary and beneficial competences and determine their appropriate combinations and sequences, especially in accordance with specific healthcare interventions.

### Target group of the real-life pilot

We have concentrated on important Hungarian population health issues and selected different sections of the Care Delivery Value Chain. We also considered the national interventional methodologies and best practices that are available. Of the seven different real-life pilot areas—(1) health planning with healthy individuals, (2) fall prevention in osteoporosis, (3) type 2 diabetes care, (4) a smoking cessation and movement program in COPD, (5) complex stroke rehabilitation, (6) cardiovascular risk assessment in school children, and (7) colorectal cancer screening and breast self-examination—we present the results of a case study of fall prevention in osteoporosis since this issue is highly pertinent, and a person-centered health management approach here may create additional health value benefits as compared to the medically driven disease-intervention approach.

For fall prevention in osteoporosis, the subject of the case study in this paper, the significance of the estimated impact is substantiated by the European Vertebral Osteoporosis Study (EVOS) according to which about 600,000 women and 300,000 men aged 50 years and over are affected by osteoporosis in Hungary [44]. The significance of osteoporosis is due to the increase in pathologic fractures and their complications, hip fractures in particular. Various surveys have revealed that 12–20% of patients with a hip fracture die in the first year after the fracture, and about half of them need care until the end of their lives, while only every fifth patient recovers fully. Most vertebral fractures occur gradually and remain hidden for long periods of time. Data from the Hungarian National Health Insurance Fund show that in 2013 only 10% of incidents were detected immediately.

Taking an integrated care approach with individualized care has been shown to enhance both rehabilitation outcomes and cost-effectiveness after hip fracture surgery [45], while person-centered care for patients undergoing total hip arthroplasty that focuses attention on patients as people and includes them as partners in healthcare decision-making can result in shorter in-patient stays [46].

## Model development

The objective of model development was to create a community-based, person-centered transaction network that manages health experiences as a potential economic model for a person-centered health service system that delivers increased value to members of the community. This section of the paper summarizes the preparatory work undertaken to create this community-based, person-centered transaction network, and the results of a real-life pilot.

The first part of the work involved a system engineering process and involved the development phases of:

- Analysis of the transaction network
- Activity sequencing
- Person-centered transaction network design

The newly engineered system was not only used as a conceptual model of the healthcare ecosystem but was implemented in a real-life pilot, involving:

- Service integration
- Individual health planning
- Community health management

Results are presented for the case of fall prevention in osteoporosis, focusing on medical achievements and improvements in health experience measures. Finally, the learning outcomes and possible implications of the Community Health Experience Model are described.

### Analysis of the transaction network

As operationalization of the Care Delivery Value Chain, the analysis of condition-specific health competency transactions in the health ecosystem was accomplished with a special focus on both patient interactions and physician activities. These transactions were organized into standardized condition-specific processes that correspond to the value chain model.

### Activity sequencing

The condition-specific value chain was fully drawn up as a continuous sequence of distinct activities. These activities represent the elements of a condition-specific transaction network.

The condition-specific value chains were broken down into the activity elements that are performed by specific actors using specific resources. These activity elements can also be regarded as constituent elements in the management of the value generation workflow.

The activity elements were classified according to the roles of the actors and then the actors into project teams according to their relations and interactions in the process.

The activity-sequence-specific matrix of actors and resources is the basis for the organization of the complex stakeholder network that supports specific care delivery value chain processes with their transactions.

**Fig. 2:**
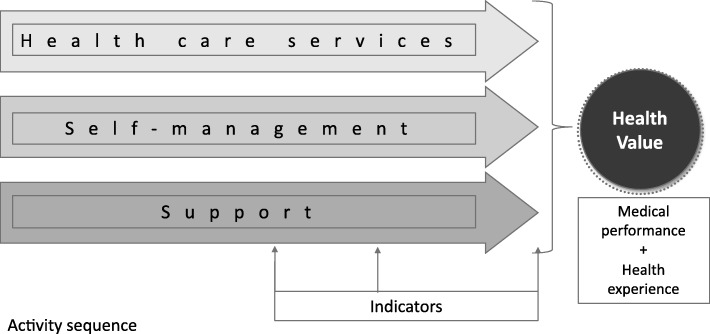
A person-centered care delivery value chain involving three transaction-specific sub-processes [26]

A flawless activity sequence (Fig. 2) that completely covers patients’ needs must consist of three sub-processes: (a) healthcare provider activity flow, (b) the patient’s own activity flow, and (c) the patient’s flow of support (which supplements the patient’s own activity when necessary).

### A person-centered transaction network

**Fig. 3:**
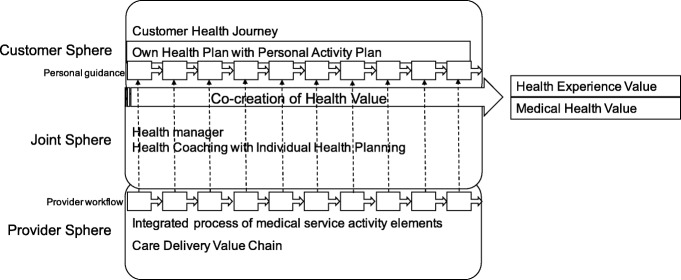
Community Health Experience Model as a conceptual framework for a person-centered health transaction network. Source: authors’ construction

We designed the value generation health ecosystem as a community-based, person-centered transaction network according to the theory of co-creation-based service logic [47], thereby emphasizing the role of value-in-use and network-based transactions. As the transaction network focuses on patients who mobilize their resources primarily according to their own health experiences [48] and the value creation activities require significant resources from the community, we call the conceptualized network the Community Health Experience Model (Fig. 3).

The model combines the Care Delivery Value Chain Model [5] with the Customer’s Own Health Plan and Health Coaching. The model serves as a conceptual model for the analysis of the complete healthcare process. The core of the model is the realization that individual effort is of the utmost importance in achieving an increase in individual health value. In this, motivation, energy, and strength are supported through positive individual health experiences. For individuals, there are three major groups of competences that facilitate value-in-use value generation:

1. Competences that individuals have learnt and acquired as part of their health literacy
2. Competences that individuals receive from the service provider sphere as customers
3. Competences that individuals receive from experts and other experienced people, typically online through non-financial exchanges

Within the framework of individuals’ health behavior and attitudes, positive or negative lived experiences and their extent considerably affect the use of competences. The more positive the lived health experience, the more the competence is integrated into everyday health-related activity.

The interconnection and interaction between individual health competence spheres results in a network of co-creational community-based health spheres. These function optimally and efficiently if the whole co-creational sphere, together with its components—customer (i.e., patient) sphere, joint sphere and service provider sphere—operate as a uniform set of competences. This uniform competence sphere allows each competence to be employed to create value-in-use by individuals and thus increases overall value, in part through synergistic effects. These co-creational spheres are connected to the standard parts of the health value increasing value chain (for example, fracture preventive health management after the first fracture in women with osteoporosis).

The health counselor, working as a representative of a new profession in a joint sphere, plays an essential role in facilitating coordination between the customer sphere and the provider sphere. Using individual health planning tools, the health counselor develops a functional project plan in which the following are defined: the linkage between the provider sphere and the customer sphere, processes to be implemented by the two actors, and the co-creational tools provided by the service provider either directly (e.g., group exercise) or indirectly (e.g., creation of a closed online forum, or providing gym membership for free).

The role of health counselor was developed by merging the two health-related activities of a case manager and a health coach. The health counselor acts primarily as a case manager in the provider sphere and provides support in terms of managing the service plan. These actors also have a role as coaches in the customer sphere by facilitating the self-management processes of customers in the achievement of their goals.

Health planning also has a role in raising individual awareness. Our health-related activities have a fixed starting point and a set goal (or goals) for each jointly defined phase. The co-creational learning process for service providers takes place primarily through the knowledge they acquire from feedback about health experiences and (thereby affected) medical outcomes, while the co-creational process facilitates the practical implementation of customer-centeredness. From a service provider’s point of view, health counseling based on individual health planning and the co-creational sphere organized thereby tends to support a positive health experience; at the same time, from the perspective of the customer sphere, the professional foundation is strengthened.

### Operational measures

The whole co-creational sphere and all the community-based health spheres must be organized to bring the model into operation: it is unrealistic to expect the emergence of a self-organized arrangement. It is useful to delegate a manager to each of the three co-creational sub-spheres to organize and manage the model:

- A professional manager with a medical degree to manage the provider sphere
- A senior health counselor with a counseling or coaching qualification to manage the community-based sphere
- A social manager to organize and manage the framework of the customer sphere

Furthermore, a community health planner should be employed to carry out and support measurement, analysis, and planning. Additionally, a business administrator should ensure sustainability and create a uniform financing framework.

### Measurement

The basis of measurement and management is the assessment of composite health-value indicators in line with the healthcare value chain, while the associated follow-up in changes in health value is demonstrated by medical indicators and health experience indicators. A uniform measurement and management framework can ensure that, instead of fragmented and rigid processes, the healthcare provider sphere can provide customers with network-type and flexible person-focused interactions that are implemented through professional teamwork. In the customer sphere, through feedback about interaction-related outcomes the measurement and management framework represents an improved guideline for individuals about the effects of competence transactions, meaning they will more carefully consider and take medical issues into account in their decision making.

## Real-life pilot

A community health transaction network was designed and developed that was appropriate for the realization of the described model.

1. Condition-specific “job-to-be-done” types of needs were defined. These involve the experience-based goals of customers and help in the establishment of a co-creative platform.
2. The next step was to define the condition-specific joint sphere, provider sphere, and customer sphere, including members of the provider team, customers in a similar condition, and supporters of value-in-use value generation.
3. By integrating health counselors and community-based healthcare managers, we incorporated new resources into the current system of healthcare resources, using current expertise and skills extensively.
4. The cumulative value creation process was supported by health counseling sessions.
5. During counseling sessions, positive customer experiences were strengthened to enhance perceived value.
6. Value-in-use value generation was facilitated by planned activities in local co-creational spheres organized by community-based healthcare managers.
7. Online creative platforms were created and used: additionally, co-creative condition-specific group sessions with moderators were developed and organized.
8. The independent co-creational activities of customers were encouraged.
9. By introducing health counseling, new resources were generated to support the everyday activities of customers.
10. Health counselors and community-based healthcare managers helped to involve healthcare providers into the health-value creation processes of customers.
11. With the coordinated and goal-oriented activities of the extended provider team, the co-creative platform was strengthened, making it capable of influencing customer value creation actively and directly.

### Service integration—transactions in the provider sphere

The activity elements of fall prevention in osteoporosis as competency transactions were first determined. Then, activity-related actor and resource competencies were established along with the patient-centered workflow with associated decision points and timing.

In the pilot, fall prevention exercises were selected as the intervention. Physiotherapists were centrally trained; an educational brochure was designed, printed, and distributed; and a fall-prevention exercise-based group work for patients was designed.

### Individual health planning—transactions in the joint sphere

Health coaching was implemented alongside individual health planning and resulted in the individual activity sequences. This process harmonized the personal needs, desires, and access to the medically designed activity elements.

The value-in-use concept was reinforced with a description of activities that were deemed the patient’s own responsibility.

### Community health management—transactions in the three spheres

The provider’s facilitating activities were organized through a community health management team. Their responsibilities consisted of capacity planning, access management, the supervision of health coaches, and reporting, along with implementing experience-sharing methods.

### Sample and duration

*N* = 20 locations in Hungary

*N* = 84 GPs

*N* = 53 Physiotherapists

*N* = 932 Female participants

The follow-up period was 3 months (from July 2015 to October 2015).

### Measurement

A fall risk assessment score was provided by the Hungarian National Institute of Rheumatology and Physiotherapy. Methodology from GfK, a market research company, was used for the measurement of health experience and consumer experience, and a questionnaire about the curative experience was drawn up by the present authors. Analysis was undertaken using IBM SPSS Statistics 24 using a Macintosh program. ANOVA analysis was also used for the determination of means and frequencies.

The National Scientific and Ethical Committee of the Medical Research Council approved the pilot, and all participants filled out and signed the appropriate informed consent form.

### Availability of data and materials

The data that support the findings of this study are available from National Healthcare Services Center in Hungary, but restrictions apply to the availability of these data, which were used under license for the current study, and so are not publicly available. However, data are available from the authors upon reasonable request and with permission from the National Healthcare Services Center.

## Results

### Medical

The risk of falling as assessed by the Hungarian National Institute of Rheumatology and Physiotherapy fall risk assessment score decreased in 11.8% of patients, of whom 5.7% from a moderate to small risk, 4.0% from a high to moderate risk, and 2.3% from high to small risk. The number of falls decreased in 4.5% of patients.

The trial carried out with patients who had been diagnosed with osteoporosis showed that a model of community-based health experience can deliver more positive outcomes in treating endemic health problems.

According to our health economy impact model, and based on data from the National Health Insurance Fund [49], the expected probability of fractures in the group of non-enrolled patients is 3% over a 6-month period, and for enrolled patients 2.3%.

Both the cost and the number of fractures were lower in the treated cohort, and results were more encouraging in the collaborative patient group. If the level of care and collaboration achieved in the trial could be generally extended, cost reductions would be further facilitated. In the collaborative group, the probability-adjusted cost of inpatient treatment for fall-related fractures was 66.86 euros per patient for 6 months, whereas in the non-collaborative group costs amounted to 88.35 euros. Using the difference of 21.49 euros, we calculated the costs for the entire population of patients with osteoporosis for a 5-year period. Results indicate that they can be decreased substantially. A total of 54,701 patients with two fractures are estimated for the entire patient population in a 6-month period, so multiplying the number of patients by the cost saving per patient, we arrive at savings of 1,175,524 euros over a 6-month period.

For the whole population, savings in 1 year amount to 2,351,048 euros, and for 5 years, 11,755,240 euros.

### Health experience measured with patient questionnaire

Self-rated condition-specific health literacy increased from 7.85 to 8.26 (0.41 improvement) (*p* = 0.05).

Self-rated condition-specific self-management capability changed from 7.25 to 8.06 (0.81 improvement) (*p* = 0.05).

### Change in consumer experience

Experience with general practitioner visit (*p* = 0.001)

**Table:** 

Memorable positive	from 31.5 to 60.3%
Memorable negative	from 41.1 to 20.5%

Experience with nurse or general practitioner (*p* = 0.05)

**Table:** 

Memorable positive	from 64.4 to 54.8%
Memorable negative	from 4.10 to 19.2%

Experience of fall prevention home exercises (*p* = 0.02)

**Table:** 

Memorable positive	from 26.0 to 34.9%
Memorable negative	from 5.5 to 12.8%

Experience of physiotherapist-led group work (*p* = 0.01)

**Table:** 

Memorable positive	from 27.4 to 42.5%
Memorable negative	from 6.8 to 15.1%

### Curative experience of general practitioners

From 22.37 (74.6%) to 23.58 (78.6%) from the maximum of 30 points awarded to six questions related to Word of Mouth, Therapy Adherence and Patient Quality of Life (Additional file 1).

## Discussion

The pilot results indicate promising outcomes for the Community Health Experience Model as regards the provision of person-centered and co-creation-based health services.

Data about medical outcomes concerning the reduction in fall risk and fall reduction are more favorable than the present approach indicates [50]. According to such health economy modeling, the nationwide realization of a person-centered community-based health service system could save 2.35 million euros annually in Hungary, which could be reallocated to patient-centric quality improvements in primary care.

The value-in-use focus of the concept and the thoroughly managed co-creation processes at the stage of implementation are reflected in all of the perceived value elements that were assessed.

Patient empowerment was highly successful in this short period of time in terms of improvements in self-rated health literacy and self-management capability. As the co-creative program focused on activities, self-management capability improved more. The facilitation of self-management and support for customer independent co-creational activities were so successful in the program that some groups decided to continue the group work after finishing the pilot period.

Improvements in customer experience were also indicated, however, to different degrees and in different patterns. The clear winners of the new service design are general practitioners. Following intervention, the proportion of memorable positive interactions doubled and memorable negative interactions were halved. Teamwork and the extra competencies supplemented with additional care and attention from health coaches reinforced physician competencies.

However, in the case of practice, nurses’ positive memorable customer experiences decreased by 10%, and negative memorable experiences increased by 15%. The reason may be that the former were the only participants from the service provider team who did not receive special training and who had little information about the new opportunities. Patients were encouraged to manage themselves; consequently, their questions changed both in terms of number and content, and nurses may not have been prepared to answer them. This experience taught us that when services are expanded and are accompanied by diversification of competences, training and information should be provided for every participant.

Changes in the perception of fall prevention home exercises and physiotherapist-led group work are clearly reflected in the increased activity requirements for patients. Although the proportion of positive memorable interactions increased, especially for group work, memorable negative experiences also increased. The growth rate of positive memorable experiences of home-based exercises was much lower than that for the group exercises (9 vs. 15%, respectively) while the growth rate of negative memorable experiences was similar in both cases (7 vs. 8%, resp.). The reason may be that, apart from sharing experiences with others, participating in group exercises effectively stimulated participants, while the user experience with the training booklet about fall prevention was only moderately positive. Although the training booklet was prepared by a highly qualified professional team, it was not found to be as useful as the other, mainly online informational materials that were employed in the program. With active contribution, memorability significantly increased, reflecting the improved effect of the specific experience. Success with this approach suggests the opportunity for the patient as actor to generate the necessary value-in-use.

Curative experiences with general practitioners improved slightly during a short period of time (+ 5.3%), presumably because the former were enabled to offer real solutions and care to their patients, and gradually experienced the positive impact of the fall prevention exercises together with the improved patient experience. Physicians expect that the extended team work that was organized for their patients and which supports their work will further improve working satisfaction.

The survey results indicate the following about the proposed Community Health Experience Model:

1. It represents a promising conceptual framework for an individual-centric system of health competence networks whereby health value-in-use is generated by network activities.
2. The scope of health competences used by the individual should be described in terms of the competences of the entire health ecosystem, which is much larger than the scope of competences available in healthcare alone.
3. The holistic approach, which involves taking the whole human being into consideration, is encapsulated in the competence network, as defined by the model, in such a way that health experience factors and corresponding indicators are integrated. In contrast to previous integrated healthcare systems, the primary development is the integration of health experiences typical of the entire health ecosystem into the model. Accordingly, the community-based health experience model, from the standpoint of healthcare systems, can be regarded as a model of health experience augmentation.
4. Health-related tasks, which are perceived and accomplished by individuals, are defined in the condition survey and goals are recorded in the health plan; such tasks can be performed alone by the individuals in the individual customer sphere, with assistance in the joint sphere, or perhaps in the service provider sphere without the consent of the individual. The activity elements and health-related goals of the individual health plans are derived from the activity chains of the healthcare value chain; thus, health-related tasks correspond to a well-defined component of the healthcare value chain.
5. The healthcare value chain that corresponds to the health-related tasks defines the “to-be-done” activity elements, which are designed to create health value, as well as the corresponding competences needed in the customer sphere, the joint sphere, and the service provider sphere. The service provider process and the customer journey that lead to health value generation are implemented in collaboration with competences in co-creation.
6. Online and offline customer spheres—linked to health-related tasks and in which health experience is the main driver of transactions—can increase the number and frequency of individually initiated connections to the key competences of the service provider sphere through the joint sphere.

Our results suggest a possible path for developing the Hungarian health care system, describing a detailed method for a reengineering process that is able to improve system efficiency. The current system is highly inefficient, since it [1] is strongly hospital-centric with large regional and societal differences in access to appropriate care [2], is highly disintegrated with huge deficiencies in the care continuum that cause significant health value losses, and [3] involves a pure customer focus, thereby lacking opportunities for patient involvement and proper treatment adherence [51].

The above-described organizational steps of service integration, individual health planning, and community health management offer a means of stepwise system improvement with the promise of care quality development with corresponding outcome development. Based on value chain logic and with a related person-centered health value generation focus, system reengineering should first focus on selected conditions and/or the risk factors that are the most compelling, like cardiovascular diseases and the most frequent types of cancer. It should also cover the complete reorganized care delivery value chain from monitoring and screening to rehabilitation and management.

## Conclusions

Application of the community health spheres approach, designed according to the person-centered competency network of the Community Health Experience Model, may improve the efficiency of the generation of health value-in-use in all three spheres (customer/patient sphere, provider sphere, joint sphere) of health generation. In the providers’ sphere, the network organization of competencies determined by the care delivery value chain, corresponding to the activity-related medical evidence, can improve both the efficiency of value generation and the curative experience of professionals, thereby increasing the satisfaction level of healthcare workers and contributing to improved customer satisfaction. The cost of the effective use of competencies may be reduced, enabling the redistribution of resources for competencies currently not accessible for health generation in any sphere that would further improve the total health value of the population.

The formal design of the joint sphere ensures that the specific sections of the provider workflows are regulated according to professional healthcare guidelines and that elements of customer journeys (driven by individual beliefs about health and health experiences) will be realized jointly and in a concerted way, generating the maximum improvements in health value-in-use.

With regard to the complete health ecosystem, the largest loss of utility is caused by disharmony and conflict between the competencies accessible inside and outside of the healthcare system. Physicians commonly claim that patients are uninformed and do not follow their recommendations, are obstinate, and fall for all kinds of quackery [30], while customers experience that physicians do not listen to them and/or simply prescribe medicine, and they find it hard to get any guidance about the potential benefits of the proliferating number of healthy options that are not officially included in the healthcare system [31].

The integration of the customer sphere into the complete health generation sphere would represent the greatest development in health value generation. We accept that many known illnesses are not curable with current technology and that health maintenance is primarily the responsibility of each individual. A community and social network approach would provide the most effective support. It would also modify the scope of public health activities, shifting the focus from behavior change intervention to providing opportunities for change by creating community health spheres. Better design of the online spheres that organically join the provider sphere and the joint sphere may also be a means of further development.

Further studies are needed to assess the large-scale manageability and sustainability of this model.

## Additional file

Additional file 1: Curative Experience Questionnaire. (DOCX 13 kb)

## References

[1] Pine BJ, Gilmore JH. Experience economy. Boston: Harvard Business Review Press; 2011. ISBN 978-1422161975.

[2] Achrol RS, Kotler P (2012). Frontiers of the marketing paradigm in the third millennium. J Acad Mark Sci.

[3] Christopherson GA. “Person-Centred” Rather Than “Patient-Centred” Health. http://content.healthaffairs.org/content/29/8/1489.short/reply#healthaff_el_450117. Accessed 4 Aug 2010.

[4] Gerteis M, Edgman-Levitan S, Daley J, Delbanco TL. Through the patient’s eyes: understanding and promoting patient-centred care. San Francisco: Jossey-Bass Publishers.

[5] Porter ME, Teisberg EO. Redefining health care; creating value-based competition on results. Boston: Harvard Business School Press; 2006. ISBN13 978–1–59139-778-6.

[6] Healthcare Intelligence Network. Benchmarks in value-based healthcare. 2014 http://www.hin.com/Presentations/Value_Based_Healthcare_SlideShare.pdf.

[7] Kaiser LS, Lee TH. Turning value-Based health care into a real business model. Harvard Business Review. 2015. p 6.

[8] Ekman I, Swedberg K, Taft C, Lindseth A, Norberg A, Brink E (2011). Person-centred care—ready for prime time. Eur J Cardiovasc Nurs.

[9] Pirhonen L, Olofsson EH, Fors A, Ekman I, Bolin K (2017). Effects of person-centred care on health outcomes—a randomized controlled trial in patients with acute coronary syndrome. Health Policy.

[10] Coulter A, Entwistle VA, Eccles A, Ryan S, Shepperd S, Perera R (2015). Personalised care planning for adults with chronic or long-term health conditions. Cochrane Database Syst Rev.

[11] Fors A, Ekman I, Taft C, Björkelund C, Frid K, Larsson ME, Thorn J, Ulin K, Wolf A, Swedberg K (2015). Person-centred care after acute coronary syndrome, from hospital to primary care—a randomised controlled trial. Int J Cardiol.

[12] Fors A, Gyllensten H, Swedberg K, Ekman I. Effectiveness of person-centred care after acute coronary syndrome in relation to educational level: subgroup analysis of a two-armed randomised controlled trial. Int J Cardiol. 2016;221:957–62. 10.1016/j.ijcard.2016.07.060. 27441475

[13] Åkerman CR, Stowell C. Measuring outcomes: the key to value-based health care. Harvard Business Webinar. 2015.

[14] Pelster M, Hagemann V, Laporte Uribe F (2016). Key aspects of a sustainable health insurance system in Germany. Appl Health Econ Health Policy.

[15] Vargo SL, Lusch RF. It’s all B2B… and beyond. Toward a system perpective of the market. Ind Mark Manag. 2011;40:181–87.

[16] Frow P, McColl-Kennedy JR, Payne A (2016). Co-creation practices: their role in shaping a health care ecosystem. Ind Mark Manag.

[17] Wismar M, McQueen D, Lin V, Jones CM, Davies M. Intersectoral governance for health in all policies. Eurohealth. 2012;18: 4.

[18] GfK Roper. Consulting trend key 3.0. Overview, Global Trends & High Impact Data. 2011.

[19] Reblin M, Uchino BN (2008). Social and emotional support and its implication for health. Curr Opin Psychiatry March.

[20] Puchalski CM (2001). The role of spirituality in health care. Proc (Bayl Univ Med Cent).

[21] Sørensen K. Health literacy: a neglected European public health disparity. Dissertation. 2013. https://cris.maastrichtuniversity.nl/portal/files/1046291/guid-fec5580d-8b4e-4855-b023-a70b4d8decba-ASSET3.0.

[22] Forget G, Lebel J. An ecosystem approach to human health. Int J Occup Environ Health. 2001;7(2):38. 11387989

[23] Ortiz-Ospina E, Roser M. ‘Financing healthcare’. Published online at https://OurWorldInData.org. Retrieved from: https://ourworldindata.org/financing-healthcare/.

[24] Lantos Z, Simon J, Bacskai I. Branding in the pharmaceutical industry. Budapest: Pirulakatedra Brochures, Marketingpirula Kft; 2006. p. 54.

[25] Vargo SL, Lusch RF (2004). Evolving to a new dominant logic for marketing. J Mark.

[26] Lantos Z. Csak egészség legyen! – De hozzá milyen egészségügy?? IME XIII. 2014. p. 9–14.

[27] Co-creating health, Briefing May 2008, The Health Foundation. http://www.health.org.uk/public/cms/75/76/313/551/Co-creating.health.briefing.paper.pdf.

[28] GfK Roper. Asthma self-care. Ethnographic research. 2008

[29] GfK Healthcare. COPD care and self-care. Ethnographic research. 2011.

[30] GfK Hungária. Health care confidence index 2010-2013.

[31] GfK: Health economy monitor, 2011–2012.

[32] GfK. The Hungarian health behaviour segments. 2012.

[33] Coleman J. Lifelong learning is good for your health, your wallet, and your social life. Harvard Business Review. 2017.

[34] Porter ME (2010). N Engl J Med.

[35] Glouberman S, Mintzberg H (2001). Managing the care of health and the cure of disease—part I: differentiation. Health care management review. Winter.

[36] Glouberman S, Mintzberg H (2001). Managing the care of health and the cure of disease—part II: integration. Health care management review. Winter.

[37] Gawande A. Health care needs a new kind of hero. Harvard business review. 2010;88(4):60-61. 20402056

[38] McDonald PA, Mecklenburg RS, Martin LA. The employer-led health care revolution. Harvard business review. 2015;93(7-8):33–50. 26540959

[39] NHS. Multispecialty community provider vanguards. 2015. https://www.england.nhs.uk/ourwork/futurenhs/new-care-models/community-sites/.

[40] Marsh K, Caro JJ, Hamed A (2017). Amplifying each patient’s voice: a systematic review of multi-criteria decision analyses involving patients. Appl Health Econ Health Policy.

[41] Ali L, Fors A, Ekman I (2018). Need of support in people with chronic obstructive pulmonary disease. J Clin Nurs.

[42] Adams J, Bakalar R, Boroch M, Knecht K, Mounib EL, Stuart N. Healthcare 2015 and care delivery. Delivery models refined, competencies defined. IBM Global Business Services, IBM Institute for business value. 2008. p.32.

[43] Jansson I, Fors A, Ekman I, Ulin K (2018). Documentation of person-centred health plans for patients with acute coronary syndrome. Eur J Cardiovasc Nurs.

[44] Poór G, Kiss C, Szilágyi M, Mituszova M, O'Neill TW, Felsenberg D, Silman AJ (1997). Prevalence of vertebral deformity in Hungary: the European Vertebral Osteoporosis Study. Orv Hetil.

[45] Olsson LE, Hansson E, Ekman I, Karlsson J (2009). A cost-effectiveness study of a patient-centred integrated care pathway. J Adv Nurs.

[46] Olsson LE, et al. Person-centred care compared with standardized care for patients undergoing total hip arthroplasty—a quasi-experimental study. J Orthop Surg Res. 2014;9(1) 10.1186/s13018-014-0095-2. 10.1186/s13018-014-0095-2 PMC4222396 25359278

[47] Grönroos C, Gummerus J (2014). The service revolution and its marketing implications: service logic vs service-dominant logic. Manag Serv Qual.

[48] Doyle C, Lennox L, Bell D. A systematic review of evidence on the links between patient experience and clinical safety and effectiveness. BMJ Open. 2013. 10.1136/bmjopen-2012-001570. PMC3549241 23293244

[49] Bacskai M, Érsek K, Somfay I. A “TÁ- MOP-6.2.5-B-13/1-2014-0001 Szervezeti hatékonyság fejlesztése az egészségügyi ellátórendszerben – Területi együttműködés kialakítása” című projekt modellezéssel megvalósított hatásvizsgálata, 2015. 2015. ÁEEK

[50] Gillespie LD, Robertson MC, Gillespie WJ, Sherrington C, Gates S, Clemson LM, Lamb SE (2012). Interventions for preventing falls in older people living in the community. Cochrane Database Syst Rev.

[51] A magyar egészségügyi rendszer teljesítményértékelési jelentése 2013-2015 (Health system performance assessment 2013–2015 report, Állami Egészségügyi Ellátó Központ, Egészségügyi Rendszer Teljesítményértékelési Munkacsoportja, Budapest. 2016. p.1142.

