# Supplementary material for: The Community Health Experience Model—value generation from person-centered health transaction network
Source: Public Health Rev. 2018 Oct 1;39:29. doi: 10.1186/s40985-018-0105-8 (PMC6166292; doi:10.1186/s40985-018-0105-8)
Supplement: Supplementary file 1 — Curative Experience Questionnaire. (DOCX 13 kb) [file 40985_2018_105_MOESM1_ESM.docx]

Curative Experience Questionnaire

Please, evaluate each statement according to your own experience, where

1=totally disagree, 2=disagree, 3=both agree and disagree, 4=agree, 5=totally agree

My patients

- recommend me to their acquaintances. 1 2 3 4 5
- live as active as non-ill people. 1 2 3 4 5
- can live without limitations. 1 2 3 4 5
- have good therapy adherence. 1 2 3 4 5
- can stay active as long as possible. 1 2 3 4 5
- come regularily back, so they are well controlled. 1 2 3 4 5
